# Entrepreneurship Education, Psychological Cognition, and Entrepreneurship Activities: An Analysis Based on a Fuzzy-Set Qualitative Comparative Analysis

**DOI:** 10.3389/fpsyg.2021.733319

**Published:** 2021-10-28

**Authors:** Yujia Jiang, Guobiao Li, Xu Cai, Zihan Yang, Yangjie Huang, Ling Zhang, Leilei Huang

**Affiliations:** ^1^Institute of China Innovation & Entrepreneurship Education, Wenzhou Medical University, Wenzhou, China; ^2^Jing Hengyi School of Education, Hangzhou Normal University, Hangzhou, China

**Keywords:** entrepreneurial education, entrepreneurial intentions, entrepreneurial opportunity identification, entrepreneurial competitions, fsQCA

## Abstract

At present, research in the field of college students' entrepreneurship has proliferated, but these studies tend to analyze the net benefits of various factors on entrepreneurial activities, which are affected by the configuration effects of multiple factors; hence, it remains unclear whether entrepreneurial education can make graduates more efficient to started their own companies. To fill this gap in the literature, drawing on general systems theory and using fuzzy-set qualitative comparative analysis (fsQCA), we take 1,87,914 undergraduate and junior college students from 1,231 colleges and universities in China as a sample to explore the relationships among the five conditions in the entrepreneurship education environment and cognitive level (i.e., the quality of staff, subject curriculum, entrepreneurial competition, intentions, and opportunity identifications) and entrepreneurial activities. The fsQCA results show that none of these factors are sufficient for entrepreneurial activity. In contrast, three combinations of the five conditions (i.e., co-creation type, competition-oriented environment, and entrepreneurship education that fits under the guidance of entrepreneurial intention) can produce high entrepreneurial activity, as well as substitution and complementarity among the various elements within the configuration. These results show that the combined effect of the five conditions is more conducive to the entrepreneurial activities of college students. Finally, after a discussion of the study's findings, theoretical, and practical contributions are analyzed with regard to the field of entrepreneurship in Chinese colleges, and alternative options indicate that college students are more likely to become entrepreneurs in the future.

## Introduction

According to a recent report released by the International Labour Organization (ILO) of the United Nations, since the COVID-19 pandemic, more than 470 million people worldwide are unemployed or underemployed. The lack of work to maintain a decent livelihood may lead to poverty, aggravate inequality, and cause social unrest (International Labour Organization, 2020). The annual Global Competitiveness Report issued by World Economic Forum has consistently shown that entrepreneurship plays an irreplaceable role in increasing employment, promoting economic development, narrowing the gap between the rich and the poor, and maintaining social stability. The single best predictor of actual entrepreneurship is entrepreneurial intention (Krueger et al., 2000). Existing research shows that entrepreneurship education and individual psychological cognition have a positive effect on entrepreneurial intention. However, using entrepreneurial intentions to predict individual entrepreneurial behavior has major limitations (Esfandiar et al., 2019), the transformation of entrepreneurial willingness to entrepreneurial behavior is still distant (Gelderen et al., 2015; Gielnik et al., 2015). Therefore, improving individual entrepreneurial ability through education and transform entrepreneurial intentions into behavior requires further investigation.

Fayolle et al. (2019) showed that the effects differ depending on trainees' personal characteristics and training strategies, which can have varied impacts on learning processes and results. On the one hand, resources are increasingly being devoted to entrepreneurship education because this will lead to a new generation of entrepreneurs (Rauch and Hulsink, 2015). Additionally, entrepreneurship education provides a platform for the common development of equal opportunities and continuous self-discovery of self-motivated potential, which are the largest sources of economic growth in the world—especially in the United States—and lays the foundation for improving entrepreneurial skills and increasing the entrepreneurial success rate (Acs et al., 1998). Although the research results support the conclusion that entrepreneurship education has an impact on students' tendencies and intentions, deficiencies still exist in current research on entrepreneurship education and activities (Pittaway and Cope, 2016). Some scholars have pointed out that entrepreneurship education has not yet fully developed, warning scholars not to fall into the trap of maturity, complacency, and stagnation, indicating that they should continue to improve and conduct more in-depth research on the subject (Solomon et al., 2002; Kuratko et al., 2005). Particularly, previous research has not provided conclusive evidence that the success of entrepreneurship is the result of participation in entrepreneurship education (Seikkula-Leino, 2008).

On the other hand, entrepreneurs' psychological cognition includes entrepreneurial intentions and opportunity identification. Although individuals with weak financial and social capital face huge challenges in identifying and developing entrepreneurial opportunities, leading to greater social inequality (Lim et al., 2016), entrepreneurial intentions and opportunity identification can still play an important role in stimulating entrepreneurial activities (Krueger, 2000; Alvarez et al., 2013). First, entrepreneurial intention is aimed at planned behavior and is the only effective predictor of entrepreneurial behavior (Krueger, 2000). Meanwhile, the identification of entrepreneurial opportunities has a positive impact on entrepreneurial intentions. It is the core resource for entrepreneurial companies to generate sustainable competitive advantages (Alvarez et al., 2013), as it is the precursor of entrepreneurial behavior (Ozgen and Baron, 2007), and can bring returns to entrepreneurs (Lumpkin and Lichtenstein, 2005). Although studies on these two aspects of research have achieved fruitful results, shortcomings still exist. In the case of entrepreneurial opportunity identification, when an individual has a strong entrepreneurial motivation or opportunity identification ability, it is difficult to produce entrepreneurial behavior without other conditions such as practical solutions (He and Zhang, 2020). For entrepreneurial intention, research has focused on its precautionary mechanism model and explores the predictive effect of entrepreneurial intention on behavior. Meanwhile, these studies have insufficiently explored the interdependence of other factors (Liñán and Chen, 2010; Schlaegel and Koenig, 2013), focusing on the independent net effect of each factor on entrepreneurial behavior but do not consider its interdependence with other factors (Shirokova and Bogatyreva, 2016).

Existing research has been limited to the independent net effect of a certain level of entrepreneurial activity, but according to the general systems theory (Boulding, 1956), the causes and conditions of social phenomena are mostly interdependent. Therefore, to explain them, a holistic and combined approach must be adopted (Ragin, 2008). The entrepreneurial system is complex; thus, entrepreneurship is a complex process affected by the synergy between individual and contextual factors (Shane and Venkataraman, 2000; Lim et al., 2016). Multiple concurrent factors affect entrepreneurial behavior. Research should explore the common results achieved through multiple factors for more accurate findings to uncover a path that stimulates individual entrepreneurial activities. In recent years, qualitative comparative analysis (QCA) has become an important tool in the fields of management, marketing, and management information systems, among others, to deal with large samples and analyze complex configuration problems (Fiss, 2007, 2011; Misangyi et al., 2017). Traditional regression analysis uses marginal analysis techniques of economics to find the optimal equilibrium (Du and Jia, 2017), while QCA uses a holistic perspective to carry out comparative analyses at the case level. Each case is regarded as a configuration of conditional variables (Rihoux and Ragin, 2009), which is more in line with the actual situation in which it is not enough to attach importance to a single factor in developing a complete explanatory model (Shaver and Scott, 1991). Thus, this study adopts the fuzzy-set qualitative comparative analysis (fsQCA) method to analyze the synergistic effects of the antecedent conditions that affect entrepreneurial activities and explore ways to improve the entrepreneurial vitality of college students, making the research results more comprehensive and feasible. Based on the above analysis, this study aims to break the existing research conclusions that examine the net effect of each influencing factor in isolation. We use theoretical analysis and configuration theory to identify the relationships among five factors (subject curriculum, entrepreneurial competitions, quality of staff, entrepreneurial intentions, and opportunity identification) and entrepreneurial activity. The factors are integrated into a research framework, and then the data are collected and sorted. Using fsQCA, the configuration effects of the five factors on individual entrepreneurial activities are analyzed, and how multi-factor concurrency can produce effective entrepreneurial activity paths to bridge the deficiencies of existing research.

In what follows we first develop the theoretical arguments and proposed the study model. Next, we report the study methodology and the results from fsQCA. We then discuss our findings and their theoretical and policy implications and conclude with some directions for future research.

## Theoretical Background and Conceptual Model

### The Role of Entrepreneurship Education

Entrepreneurship education is a process of cultivating individuals' comprehensive qualities, such as entrepreneurial awareness, thinking, and skills, so that one possesses the knowledge and skills required for entrepreneurial activities (Jones and English, 2004). Drucker (1985) believes entrepreneurship to be its own unique subject, and entrepreneurial ability can be obtained through learning. With the deepening of entrepreneurship research, further research on entrepreneurship education has also been proposed. Turker and Selcuk (2009) found that if schools provide students with correct examples in entrepreneurship education and material support, the possibility of students choosing to engage in entrepreneurship increases. The holistic development of entrepreneurship education is subject to comprehensive interference from many factors (Ma and Bai, 2015). Colleges and universities are the main battlefields for innovation and entrepreneurship education. These institutions should begin by allocating teachers, creating special courses, constructing practice systems, and conducting awareness training (Zhang, 2014). On the one hand, an educational institution's faculty serves as the core force of innovation and entrepreneurship education, and teachers' cognition of their own entrepreneurship education skills is closely related to the implementation of such education (Ruskovaara and Pihkala, 2013). King's College, London regularly guides its students in entrepreneurial knowledge and skills training by building a diversified team of entrepreneurial teachers, effectively promoting students to quickly enter the field of innovation and entrepreneurship, and has won unanimous praise from students for such an initiative (King's College London, 2020). There is no doubt that the development of entrepreneurship education cannot be separated from the development of entrepreneurial teachers (Huang et al., 2020b). However, teachers, as the backbone of entrepreneurship education, are at a crossroads as several transitions/processes converge in entrepreneurship education (Hytti et al., 2002; Hytti, 2004). Existing research also shows that the lack of professional innovation and teachers is the main obstacle to the development of innovation and entrepreneurship education (Dahl, 2019; Huang and Huang, 2019). Therefore, how to build a team of high-quality teachers and transform people with entrepreneurial abilities or ideas into innovative and entrepreneurial talents is worthy of in-depth study. This is of vital importance in improving the quality of entrepreneurship education and promoting social and economic development.

The quality of courses also has an important impact on the quality of entrepreneurship education. American pragmatism educator John Dewey, in his book *Democracy and Education*, divided the types of courses into subject and activity courses. Generally, subject courses impart basic theoretical knowledge and skills, and activity courses guide entrepreneurial practice. At present, entrepreneurship courses offered by universities around the world take both subject and activity courses into account, and all have a positive impact on college students' entrepreneurial activities. Franke and Lüthje (2004) and Turker and Selcuk (2009) used questionnaires to directly test the dimension of school entrepreneurship education and concluded that the possibility of entrepreneurship is positively affected by the school's entrepreneurial knowledge and the quality of entrepreneurial support provided. Stanford University provides a compulsory public course for all academics. The course emphasizes a multi-disciplinary approach, the combination of liberal arts and science, and an equal emphasis on theory and practice so that students can master the skills and methods of innovation and entrepreneurship in the course of their studies (Stanford Technology Ventures Program Courses, 2016). However, studies have shown that different entrepreneurship education organization methods have different effects on college students' entrepreneurial intentions. Practical entrepreneurship education methods are more effective than indoctrination. Practice methods such as entrepreneurial competition, business, and planning affect students' entrepreneurship learning processes to help students perceive the specific steps and complex process of enterprise, thereby increasing the possibility of undergraduate entrepreneurship (Kuckertz and Mandl, 2013). Particularly, entrepreneurial competition plays an indispensable role in such practices. There are various types of entrepreneurship competitions on at the Massachusetts Institute of Technology (MIT) campus. The most famous and prestigious MIT $100 K Entrepreneurship Competition plays an important role in inheriting MIT's innovative and entrepreneurial spirit (Massachusetts Institute of Technology, 2016). A survey conducted among students at Tsinghua University found that college students are more likely to experience the fun of entrepreneurship when participating in entrepreneurial competitions and when the possibility of entrepreneurial activities is enhanced (Xiang and Lei, 2011). Hence, in the course of such activities, this study selects entrepreneurial competition as the research variable.

### The Role of Psychological Cognitive

For developing countries, the underclass changes their social status through entrepreneurship and narrows the gap with developed countries, because individual psychological cognition (such as entrepreneurship intention and opportunity identification) plays an indispensable role in stimulating entrepreneurial activities, which has also been recognized by many scholars (Alvarez et al., 2013). Entrepreneurship intention is a mental state that guides individuals to pursue entrepreneurial goals by investing in energy and resources. It is also a self-commitment to consciously start a business (Bird, 1988; Scott, 2001; Carsrud and Brännback, 2011). The study of entrepreneurship intention has focused on the precautionary mechanism models of entrepreneurial intention. Among these, the entrepreneurial event and planned behavior model possess strong explanatory power with regard to entrepreneurial intentions. Based on the Theory of Planned Behavior (TPB), entrepreneurial intention is the key intermediate path for entrepreneurial behavior (Schlaegel and Koenig, 2013). Entrepreneurship intention willful and is the dominant, unique, and optimal predictor of entrepreneurial behavior. Individuals must influence entrepreneurial behavior through entrepreneurial intentions (Krueger, 2000).

Entrepreneurship opportunity identification refers to the ability to identify a good idea and transform it into a business concept (or make a considerable improvement to the enterprise), thereby cultivating a higher commercial or social value, and bringing benefits to entrepreneurs (Lumpkin and Lichtenstein, 2005). Opportunities refer to a positive organizational environment for individuals from which they can profit, so they are more inclined to start a business, and entrepreneurs with a high ability to identify entrepreneurial opportunities can recognize risks more easily and make more appropriate decisions. For entrepreneurs themselves, whether they can grasp the right entrepreneurial opportunities and transform them into successful enterprises through sufficient development is one of the most important abilities that they should possess. Singh (2001) proposed a three-stage opportunity-identification model. These three stages are to (1) generate the idea of establishing a company, (2) identify potential opportunities, and (3) make decisions to establish a business (Singh, 2001). This shows that the identification of entrepreneurial opportunities is the precursor of entrepreneurial activity engagement (Ozgen and Baron, 2007), and capable entrepreneurs can find attractive and valuable opportunities for execution, thereby promoting the production of such activities (Timmons and Spinelli, 2010).

**Figure 1:**
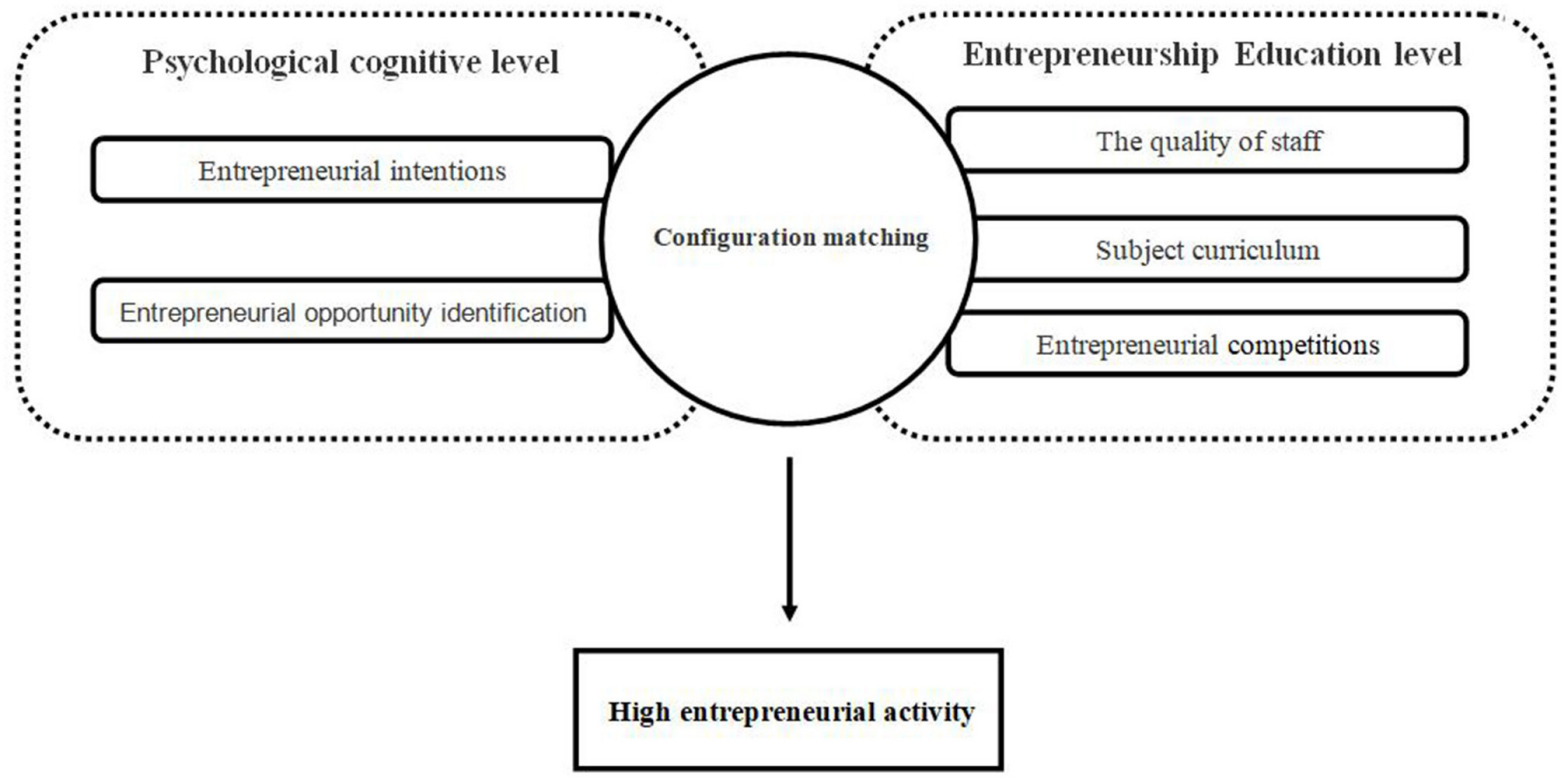
Concept model.

Based on the above analysis, this study constructs a conceptual model, as shown in Figure 1.

## Methods and Data

### Qualitative Comparative Analysis

This study adopts qualitative comparative analysis (QCA) to improve the research on the factors affecting the entrepreneurial activity of college students, aiming to explain a problem from the perspective of variance theory, i.e.„ assuming that an independent variable is a sufficient and necessary condition for the outcome. It is believed that the relationship between an independent and an outcome variable is symmetric. Moreover, in the process of explaining the difference in results, the respective variables are in a competitive relationship, not a result of a common effect. In contrast, the overall perspective of QCA is based on configuration theory (configuration is a combination of antecedent conditions), which states that a phenomenon/result should be understood as a cluster of interconnected structures and practices, rather than units or loosely integrated entities (Liu et al., 2017). At the same time, configuration theory simulates the asymmetric relationship between antecedent variables, that is, whether a certain result does or does not appear to be explained separately through different combinations of reasons (Fiss, 2011).

QCA is divided into clear-set qualitative comparative analysis (csQCA), multi-value qualitative comparative analysis (mvQCA) and fuzzy-set qualitative comparative analysis (fsQCA) according to variable types. The methods of mvQCA and csQCA are based on a clear set and truth table; they are only suitable for dealing with kind questions (Cronqvist and Berg-Schlosser, 2009). The emergence of fsQCA further improves the ability to analyze distance and ratio variables, enabling QCA to handle not only category issues, but also degree and partial membership issues; Furthermore, fsQCA, by converting fuzzy set data into a truth table, retains the advantages of truth table analysis and processing of qualitative data, limited diversity, and simplified configuration; thus, fsQCA has the dual properties of qualitative and quantitative analysis (Ragin, 2008).

In addition, fsQCA is more robust than traditional regression methods (Liu et al., 2017). On the one hand, the results of traditional regression are very sensitive to outliers, but fsQCA is not affected by outliers because its analysis relies on identifying a subset of data. Because each observation is converted into a combination of conditions, including or excluding particular data, will only change the evaluation of the combination and will not affect the overall evaluation. On the other hand, fsQCA will not be affected by the sample. This is because when fsQCA evaluates a configuration, it only considers the subset of samples in the entire dataset that is affected. If a particular configuration is expressed too much or too little, it has a minor effect on the existence of other configurations. It is because of these advantages of fsQCA that its utilization rate has increased dramatically in the field of social sciences in recent years. Therefore, this study selects the five condition variables mentioned above, including entrepreneurial intention and opportunity identification, subject courses, entrepreneurial competitions, quality of staff, and an outcome variable (i.e., entrepreneurial activity), which are analyzed using fsQCA.

### Sampling

American sociologist Charles Larkin proposed that “QCA applies to the case-oriented study of small and medium samples.” Additionally, Rihoux and Ragin (2017) proposed the principle of “selecting cases for small and medium samples,” i.e., “full homogeneity and heterogeneity in the population of cases” (Rihoux and Ragin, 2017). This study screens and integrates collected questionnaires and classifies 1,231 colleges and universities across China by province. Because the obtained sample size of Ningxia Province is too small (*N* = 2), it is not representative; therefore, only 30 provinces are examined in this study. In summary, 30 samples are included in the QCA analysis.

### Variable Measuring and Descriptive Statistical Analysis

The measurement variables used in this study regarding entrepreneurship activity, entrepreneurship intention, entrepreneurship opportunity identification, the quality of staff, subject curriculum, and entrepreneurship competition are derived from the study entitled “The Quality Evaluation of Innovation and Entrepreneurship Education: An Empirical Study from 1,231 Colleges and Universities in China.” Huang and Huang (2019) Huang's study compared and analyzed various existing innovation and entrepreneurship education questionnaires, and combined in-depth semi-structured interviews with information retrieved from several experienced teachers in this field to comprehensively design a scale for the quality evaluation of innovation and entrepreneurship education. The concept of research variables is shown in Table 1. Particularly, entrepreneurship activities and entrepreneurial intentions were measured using the percentage of indicators. The Likert scale is used to measure the adequacy of entrepreneurship opportunity identification, the quality of staff, subject courses, and entrepreneurial competitions. The higher the score, the greater the adequacy. The measurement of each variable of entrepreneurship education takes the mean value of the questionnaire items. The findings of the descriptive statistical analysis for each research variable are presented in Table 2.

**Table 1:** Variable selection and assignment.

**Variable**	**Data source**	**Secondary indicators**	**Variable definition and description**
Entrepreneurial activity	Questionnaires	—	Proportion of the number of people who engage in entrepreneurial activities during school
Psychological cognition	Questionnaires	Entrepreneurial intention	Proportion of people planning to start a business after graduation
	Questionnaires	Entrepreneurial opportunity identification	Thinking that the province's entrepreneurial opportunities are generally good
Entrepreneurship education	Questionnaires	Quality of staff	Thinking that teachers have diverse teaching methods, entrepreneurial experience, and rich entrepreneurship education experience
	Questionnaires	Subject courses	Thinking that the types of courses are diverse, the content is closely integrated with the professional knowledge, and the trends of the times
	Questionnaires	Entrepreneurial competition	Thinking that the competition types are diverse, the project and the professional are highly integrated, and it is easier to land

**Table 2:** Descriptive statistical analysis results of the research variables.

**Statistical indicator**	**Antecedent variable**					**Outcome variable**
	**Psychological cognition**		**Entrepreneurship education**			
	**Entrepreneurial intention (%)**	**Entrepreneurial opportunity identification**	**The quality of staff**	**Subject courses**	**Entrepreneurial competition**	**Entrepreneurial activity (%)**
Mean	11.18	3.02	3.42	3.36	3.36	19.33
Standard deviation	2.90	0.20	0.23	0.14	0.22	6.65
Minimum	5.00	2.54	3.04	3.13	3.00	9.30
Maximum	17.70	3.48	3.98	3.75	3.98	38.7

### Variable Calibration

Calibration refers to the process of assigning a collective membership to a case (Du and Jia, 2017). Variable calibration requires a combination of theory and practice to set three critical values: complete membership, intersection, and complete non-membership. Referring to the research of Fiss (2011), this study sets the three anchor points of five condition variables and one result variable as the upper quartile of the sample data, the mean of the upper and lower quartiles, and the lower 4th quantile. The calibration anchor points for each variable are listed in Table 3.

**Table 3:** Calibration anchor points of various variables.

**Variable**			**Target collection**	**Anchor point**		
				**Fully affiliated**	**Crossover point**	**Completely unaffiliated**
Antecedent variable	Psychological cognition	Entrepreneurial intention	High entrepreneurial intention	13.25	11.45	9.65
		Entrepreneurial opportunity identification	High entrepreneurial opportunity identification	3.14	3.01	2.87
	Entrepreneurship education	Quality of staff	High quality of staff	3.54	3.40	3.25
		Subject courses	High quality of subject courses	3.43	3.34	3.25
		Entrepreneurial competition	High quality of entrepreneurship competition	3.47	3.34	3.21
Outcome variable		Entrepreneurial activity	High entrepreneurial activity	20.8	18.14	15.48

## Results

This study uses fsQCA software to analyze the entrepreneurial activity data of 30 provinces and obtains a configuration that is determined to have high entrepreneurial activity. Following the recommendations of Fiss (2011), we set the consistency threshold to 0.8. At the same time, we refer to the recommendations of Du and Jia (2017) to set the proportional reduction in inconsistency (PRI) consistency threshold to 0.70, and the case threshold setting as 1; the case of high entrepreneurial activity is retained.

### Necessary Condition Analysis

A necessary condition analysis is required before the analysis of the fuzzy set truth table. The necessary condition is a superset of results. If it is included in the truth table analysis, it may be eliminated using a parsimonious solution (Rihoux and Ragin, 2017). The necessary conditions for entrepreneurial activities are presented in Table 4.

**Table 4:** Necessity testing of entrepreneurial activity.

**Antecedent variable**		**Outcome variable**
		**High entrepreneurial activity**
Psychological cognition	Entrepreneurial intention	0.58
	~ Entrepreneurial intention	0.52
	Entrepreneurial opportunity identification	0.55
	~ Entrepreneurial opportunity identification	0.53
Entrepreneurship education	Quality of staff	0.61
	~ Quality of staff	0.46
	Subject courses	0.71
	~ Subject courses	0.43
	Entrepreneurial competitions	0.62
	~ Entrepreneurial competitions	0.45

As shown in the table, the necessity of each item's antecedent conditions affecting high entrepreneurial activity does not exceed 0.9, which does not constitute a necessary condition; that is, the explanatory power of a single condition variable on entrepreneurial activity is weak. Therefore, these conditional scalars are included in the fsQCA analysis for the configuration of high entrepreneurial activity.

### Configuration Analysis

This study uses fsQCA3.0 to analyze the environmental configurations that lead to high entrepreneurial activity. These configurations represent different environments that achieve the same result (i.e., high entrepreneurial activity). At the same time, the configuration found in this study is named according to the configuration theory process (Furnari et al., 2020).

In this study, the raw consistency threshold is set to 0.8, the PRI consistency threshold is set to 0.7, and the case frequency threshold is set to 1. Due to the lack of evidence and theory that such conditions affect the exact direction of results, this study assumes that the presence or absence of each condition contributes to a high degree of entrepreneurial activity when conducting a counterfactual analysis. By comparing the nested relationship between the intermediate and the parsimonious solution, the core condition of each solution is identified; that is, the condition that the intermediate solution also appears in the parsimonious solution, which has an important impact on the result. The condition for the emergence of the intermediate solution is an edge condition, which is required for auxiliary contributions (Du and Jia, 2017). The QCA analysis results are presented in Table 5. There are four configurations (M1, M2, M3a, and M3b) that produce high entrepreneurial activity. Among these, M3a and M3b constitute a second-order equivalent configuration, that is, their core conditions are the same (Fiss, 2011), and the consistency indicators of the four configurations are 0.896, 0.860, 0.916, and 0.815, respectively. This shows that the four configurations are sufficient for high entrepreneurial activity. At the same time, the solution consistency is 0.880, indicating that the four configurations covering most of the cases are sufficient conditions for high entrepreneurial activity. The solution coverage of the model is 0.461, indicating that the four configurations explain approximately 50% of the reason for high entrepreneurial activity. The following is a detailed analysis of each configuration that affects entrepreneurial activity.

Table 5Configurations that produce high entrepreneurial activity.

**Table:** 

**Antecedent variable**	**High entrepreneurial activity**			
	**M1**	**M2**	**M3a**	**M3b**
Entrepreneurial intention	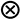	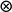	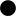	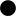
Entrepreneurial opportunity identification	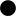	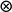	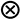	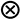
Quality of staff	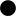	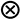	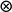	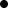
Subject courses	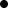	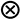	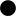	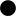
Entrepreneurial competition		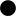	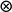	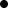
Consistency	0.896	0.860	0.916	0.815
Raw coverage	0.228	0.105	0.112	0.129
Unique coverage	0.184	0.073	0.070	0.067
Solution coverage			0.880			
Solution consistency	0.461

**Table:** 

**Attached Table 1**. Robustness test for increasing the frequency threshold.						
**Antecedent variable**	**High entrepreneurial activity (the threshold = 1)**				**High entrepreneurial activity (the threshold = 2)**	
	**M1**	**M2**	**M3a**	**M3b**	**M1′**	**M2′**
Entrepreneurial intentions	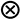	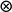	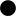	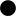	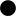	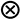
Entrepreneurial opportunity identifications	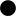	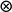	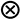	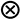	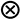	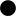
The quality of staff	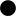	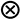	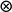	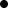	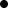	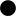
Subject courses	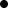	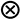	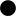	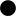	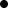	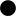
Entrepreneurial competitions		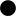	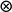	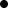	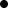	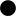
Consistency	0.896	0.860	0.916	0.815	0.815	0.890
Raw coverage	0.228	0.105	0.112	0.129	0.129	0.176
Unique coverage	0.184	0.073	0.070	0.067	0.090	0.137
Solution coverage			0.880		0.850	
Solution consistency			0.461		0.266	

#### (a). Co-creation Type

M1 shows that regardless of whether the entrepreneurial competition is good or not or if college students have low entrepreneurial intentions, as long as they have a high recognition of entrepreneurial opportunities, high-quality teachers, and a subject curriculum system, it can drive college students to invest in entrepreneurial activities. This requires schools, teachers, and students to work together. First, schools must establish and improve their innovation and entrepreneurship education curriculum systems. The entire process of entrepreneurship education is carried out around students, to cultivate students' entrepreneurial ability and spirit. Second, the quality of teachers, teachers' initiative, enthusiasm, etc. should be fully manifested. At the moment, entrepreneurial courses are mostly designed by school teachers; they possess business experience (Wu and Chen, 2019) Hence, entrepreneurial education calls for teachers with entrepreneurial experience and abilities. Finally, college students serve as the main body of entrepreneurial activities. Trait theory is used to explain the influence of individual factors on entrepreneurial intentions. Students with the ability to recognize entrepreneurial opportunities consciously perceive changes in their environment. In the process of high-quality theoretical teaching by high-quality teachers, they can combine theoretical knowledge with the opportunities that can be identified and make full use of these to inspire entrepreneurial activities. As a result, college students who can have the ability to recognize opportunities do not need to engage in competitions to build a platform to seek such opportunities, so they do not have to participate in high-quality competitions. Stanford University relies on the unique resource advantages of Silicon Valley to encourage and guide teachers and students to enter the enterprise. On the one hand, it allows students to learn, observe, and practice in the front line of the enterprise. On the other hand, it also improves the level of teachers' innovation and entrepreneurship education. To break the barriers between professional courses and innovation and entrepreneurship education courses, all college departments, including the school itself, set up such courses based on the actual development of disciplines (Stanford University, 2016), which is convenient for students of various majors to receive education in innovation and entrepreneurship theories.

#### (b). Competition-Oriented Environment

M2 shows that as long as a good competition environment is maintained, even if other conditions are at a low level, it can drive college students to carry out entrepreneurial activities. Practice in the form of entrepreneurial competitions will affect students' learning processes, help students perceive the specific steps and complex processes of enterprise entrepreneurship (Kuckertz, 2013), build a platform for college students, broaden their connections and resources, and lead to the formation of a broad business vision. The MIT $100 K Entrepreneurship Competition is the best example. The competition is held over nearly one year and is divided into an elevator speech, executive outline contest, and business plan contest. Students are free to form a team to participate, boldly develop entrepreneurial plans, and enjoy a set of resources provided by the competition's organizing committee. The winning projects of the competition can be successfully implemented in practice. The data of the relevant report show that the MIT $100 K Entrepreneurship Competition has created more than 4,600 jobs (Roberts and Eesley, 2012).

#### (c). Entrepreneurship Education Fits Under the Guidance of Entrepreneurial Intention

This is a combination of M3a and M3b. These models show that college students who possess high entrepreneurial intentions tend to be more enthusiastic about carrying out entrepreneurial activities after receiving adequate education and training. Sheeran (2002)'s meta-analysis of the relationship between willingness and behavior shows that willingness can only explain 28% of the variation in behavior. Shirokova and Bogatyreva (2016) found that the school environment has a significant regulatory effect on the entrepreneurial intention-behavior transformation process. Hence, when a person has the idea of starting a business, they also need support from the external environment to encourage them to realize their ideals. According to motivation theory, when people's subjective desire or intention to pursue a certain goal reaches a certain intensity, it needs to be transformed into motivation. According to the Theory of Planned Behavior (TPB), behaviors that are not completely controlled are not only affected by behavioral intentions but also restricted by actual conditions such as personal ability, opportunities, and resources to perform the behavior. When actual control conditions are sufficient, behavioral intentions directly determine behavior (Fishbein and Ajzen, 1975). Therefore, when college students with entrepreneurial intentions receive the required education, they will be inspired to realize the necessity of entrepreneurial activities and engage in them. The curriculum system of the Babson College Business School, known as the “basic paradigm of the curriculum of entrepreneurship education in American colleges and universities,” fits with this idea. Babson College arranges courses according to students' learning stages, which are mainly divided into three stages: discovery, exploration, and focus. In their first and second academic years, students must take introductory courses; in the second and third academic years, they are taught a series of comprehensive courses; in the third and fourth academic years, entering corporate internships and practice is mandatory, and the courses are based on strategic management and advanced liberal arts. This approach integrates entrepreneurial consciousness, personality traits, core competence, and other “entrepreneurial genetic codes” that meet the needs of future entrepreneurship and social knowledge, so that students can form an effective connection between theoretical knowledge and specific practice (Babson College, 2016).

### Robustness Analysis

This study conducts a robust test on the antecedent configuration of high entrepreneurial activity (Judge et al., 2020). First, the threshold of the number of cases increases from one to two, and the resulting configuration is essentially the same (see Attached Table 1). Second, the PRI consistency increases from 0.70 to 0.75, and the resulting configuration is consistent. The robustness test shows that the results are robust.

### Theoretical Contributions

Most existing studies explore the impact of entrepreneurship education on individual psychology and abilities, or mainly focus on the theory of planned behavior, verifying that entrepreneurial willingness is an effective indicator of predicting entrepreneurial behavior from a cognitive or motivational perspective (Schlaegel and Koenig, 2013; Kautonen et al., 2015). However, research on the relationship between entrepreneurship education, psychological cognition, and entrepreneurship activities provides a new perspective.

Nowadays, there is a certain distance between entrepreneurial willingness and behavior change; The Global Entrepreneurship Monitor reports have also disclosed that although most people possess strong entrepreneurial willingness, only a few people engage in entrepreneurial behaviors. Pittaway and Cope (2016) concluded what is unclear is the extent to which entrepreneurship education impacts on the level of graduate entrepreneurship or whether it enables graduates to become more effective entrepreneurs. Thus, this study not only explores the impact of entrepreneurship education on entrepreneurial individuals' cognition and activities but also the influencing factors and paths that truly stimulate individuals' entrepreneurial behavior.

This study provides a new methodology for research on the relationship between entrepreneurship education, psychological cognition, and entrepreneurship activities. Owing to the complexity of the system, the appearance of a certain result often does not depend on the influence of single but multiple factors. We apply fsQCA to analyze the factors that affect the entrepreneurial activities of college students, that is the configuration path between the factors, and expanded upon the theoretical research on their entrepreneurial activity. This study integrates the linkage mechanism of five variables to activate the entrepreneurial activities of college students, breaks the predicament of previous studies being limited to studying single factors, and is more in line with reality. In adopting the QCA method, the substitution and complementarity among the various elements in the configuration are also found to affect student's entrepreneurial activities. Entrepreneurship intention and the ability to identify entrepreneurial opportunities have a substitute effect, that is, when high-quality teachers and courses are available, as long as students possess a high entrepreneurial intention or opportunity recognition ability, high entrepreneurial activity can be driven. This fully embodies the advantages of QCA in interpreting the relationships between various elements within each configuration, breaking through the limitations of traditional statistical methods such as black boxes (Fiss, 2011), and providing a reference for future interpretation of this complex phenomenon.

### Practical Implications

For the current state of research, empirical research begins to show that entrepreneurship education can have an impact on the awareness and perceptions of students, where it engages them with “real-life” opportunities to learn and involves them in experiential forms of learning. There is no doubt entrepreneurship education fosters entrepreneurial intentions and thus entrepreneurial activity (e.g., Souitaris et al., 2007; Gelderen et al., 2015; Rauch and Hulsink, 2015). However, this remains a rather simplistic picture. Robb et al. (2014) consider the evidence that entrepreneurship education to shape entrepreneurship skills can effectively facilitate entry into self-employment remains thin. Recently, Chinese governmental agencies have made all-out efforts to promote school-wide entrepreneurship education from top to bottom to achieve sustainable economic and social development (Huang et al., 2020a). By drawing on general systems theory and using fsQCA, we provide empirical evidence of the effectiveness of different ways to promote entrepreneurship among students. Taking 1,87,914 undergraduate and junior college students from 1,231 colleges and universities in China as a sample, the results have important implications for academic institutions providing entrepreneurship education to promote students' entrepreneurial activity.

One of the major challenges of any economy is the promotion of entrepreneurial activity (Padilla-Angulo et al., 2021), and entrepreneurship education has been shown to have a positive impact on the intentions of young people toward entrepreneurship, their employability, and their role in society (Martin and Håkan, 2013; Bae et al., 2014; Nabi et al., 2017). In general, the greater the autonomy of students in the entrepreneurship, and the higher the quality of entrepreneurship education, the greater its positive effect on students' entrepreneurship activities. We also show that if one seeks to activate individual behaviors, one cannot rely on a single factor and must be affected by multiple antecedent configurations, which has important implications for the accurate understanding and interpretation of entrepreneurial behaviors.

First, this research shows that multi-party cooperation and joint efforts are the key forces that stimulate college students' entrepreneurial enthusiasm. To stimulate entrepreneurial behavior through entrepreneurship education, schools, teachers, and students are indispensable in the process. First, students are the main bodies of entrepreneurial activities. Only when students have an awareness of entrepreneurship and are good at discovering opportunities can such activities be carried out smoothly. Schools should consider the “student” as the center when formulating policies, carrying out training, setting up courses, establishing subsidies, etc. to improve students' enthusiasm and initiative to participate in entrepreneurial activities. Second, the quality of teachers determines the quality of education courses and directly affects entrepreneurship education. Due to the particularity of entrepreneurial education, entrepreneurship teachers also need to emphasize entrepreneurship skills (Huang et al., 2020a). At present, the dearth of highly capable entrepreneurship education teachers is the main obstacle at colleges and universities (Dahl, 2019). The fundamental problem of entrepreneurship education in colleges and universities involves teachers, as the quality of the teaching staff does not meet the needs of entrepreneurship education practice. Finally, this study also found that entrepreneurial intention and opportunity recognition ability have a substitute effect, that is, when high-quality teachers and subject courses are available, as long as students have a high entrepreneurial intention or entrepreneurial opportunity recognition ability, high entrepreneurial activity can be driven. Therefore, taking all aspects of entrepreneurship education into account, creating high-quality entrepreneurship education is more important.

Similarly, this study also shows that the entrepreneurship education curriculum plays an indispensable role in activating college students' entrepreneurial activities. To emphasize on the importance of courses, colleges and universities need to make great efforts in setting the curriculum. First, in terms of theoretical courses, colleges and universities should pay attention to the construction of innovation and entrepreneurship curriculum resources and strive to set up specialty-related general courses. Second, in terms of activity courses, attention should be paid to students' entrepreneurial practices, and various activities should be organized to enrich students' entrepreneurial activity channels and improve their entrepreneurial enthusiasm.

Finally, the power of entrepreneurial competition cannot be ignored. Entrepreneurial competition platforms can be built by relying on various forms of competition to improve students' entrepreneurial awareness, including industry-based entrepreneurial competitions in the National University Student Innovation and Entrepreneurship Training Plan, and small-scale competitions organized by schools. This can help students perceive the specific steps and complex process of business entrepreneurship, thereby improving the possibility of college students' entrepreneurship. In this way, these findings can be useful for policymakers and institutions responsible for creating training programs, as well as their inclusion in the curricular structures of the various learning cycles to influence the antecedents of entrepreneurial behavior.

## Conclusions

This study examines the data of undergraduate and junior college students from 1,231 colleges and universities in China and discusses the factors influencing entrepreneurial activities and the interaction mechanism between these factors. The study finds that it is not a single element that constitutes a necessary condition for college students' entrepreneurial activity, but a combination of three paths: co-creation type, competition-oriented, and entrepreneurship education fits under the guidance of entrepreneurial intention. Particularly, entrepreneurial intention and the ability to identify entrepreneurial opportunities have a substitute effect on entrepreneurial activity, that is, when high-quality teachers and courses are available, as long as students possess high entrepreneurial intention or opportunity recognition ability, entrepreneurial activity can be driven.

This study has the following limitations. First, many factors affect college students' entrepreneurial activities. This study only analyzes the two aspects of psychological cognition and entrepreneurship education. The selection of variables has not been sufficiently comprehensive, and entrepreneurship policies, social capital, and so on cannot be excluded. The existence of influencing factors and configuration paths must also be considered. Second, in the field of management, although qualitative comparative analysis methods have been gradually recognized, the relationship and role between this method and quantitative research method still need to be further discussed in the future. Lastly, this study is limited to the Chinese scenario, with only in-depth discussions of China. The sample range needs to be expanded to explore the factors that affect global entrepreneurial activities.

## Data Availability Statement

The original contributions presented in the study are included in the article/supplementary material, further inquiries can be directed to the corresponding author/s.

## Author Contributions

All authors listed have made a substantial, direct and intellectual contribution to the work, and approved it for publication.

## Funding

This work was supported by the Key Project of the National Social Science Fund of China – the research on barriers and policy support mechanisms for female entrepreneurship in the Digital Era (20ASH012).

## Conflict of Interest

The authors declare that the research was conducted in the absence of any commercial or financial relationships that could be construed as a potential conflict of interest.

## Publisher's Note

All claims expressed in this article are solely those of the authors and do not necessarily represent those of their affiliated organizations, or those of the publisher, the editors and the reviewers. Any product that may be evaluated in this article, or claim that may be made by its manufacturer, is not guaranteed or endorsed by the publisher.
